# Beta-Defensin-2 and Beta-Defensin-3 Reduce Intestinal Damage Caused by *Salmonella typhimurium* Modulating the Expression of Cytokines and Enhancing the Probiotic Activity of *Enterococcus faecium*

**DOI:** 10.1155/2017/6976935

**Published:** 2017-11-09

**Authors:** Alessandra Fusco, Vittoria Savio, Marcella Cammarota, Alberto Alfano, Chiara Schiraldi, Giovanna Donnarumma

**Affiliations:** Department of Experimental Medicine, University of Campania “Luigi Vanvitelli”, Via De Crecchio No. 7, 80138 Naples, Italy

## Abstract

The intestinal microbiota is a major factor in human health and disease. This microbial community includes autochthonous (permanent inhabitants) and allochthonous (transient inhabitants) microorganisms that contribute to maintaining the integrity of the intestinal wall, modulating responses to pathogenic noxae and representing a key factor in the maturation of the immune system. If this healthy microbiota is disrupted by antibiotics, chemotherapy, or a change in diet, intestinal colonization by pathogenic bacteria or viruses may occur, leading to disease. To manage substantial microbial exposure, epithelial surfaces of the intestinal tract produce a diverse arsenal of antimicrobial peptides (AMPs), including, of considerable importance, the *β*-defensins, which directly kill or inhibit the growth of microorganisms. Based on the literature data, the purpose of this work was to create a line of intestinal epithelial cells able to stably express gene encoding human *β*-defensin-2 (hBD-2) and human *β*-defensin-3 (hBD-3), in order to test their role in *S. typhimurium* infections and their interaction with the bacteria of the gut microbiota.

## 1. Introduction

The gastrointestinal tract is the most important immune organ of the human body. The intestinal surface has a strategic position at the interface between the antigenic luminal environment and the internal milieu of the host and is constantly exposed to various antigens from food or from different pathogens.

The human intestine hosts a large and diverse microbial community and contains approximatively 400–1000 different species of bacteria, virus, and fungi. These microbes are collectively referred to as the commensal microbiota.

The importance of the homeostatic maintenance of human health by the intestinal microbiota has become a topic of great interest [1–4]. Commensal bacteria modulate the expression of genes involved in several major intestinal and extraintestinal functions, including the xenobiotic metabolism, postnatal intestinal maturation, nutrient absorption, and fortification of the mucosal barrier, and inhibit the growth of pathogenic species through the production of antimicrobial substances. In addition, the human microbiota is involved in the synthesis of essential amino acids and vitamins (K, B2, B1, B6, B12, folic acid, biotin, and pantothenic acid) in the absorption of calcium, magnesium, and iron, in the extraction of energy from components in the diet, and in the regulation of fat storage [5, 6].

The genus *Enterococcus* is a group of lactic acid bacteria (LAB) whose use as probiotic microorganisms is controversial [7] as they are sometimes associated with infections in humans [8–11]. However, it has been shown that several enterococcal strains, which may rebalance the intestinal bacterial flora in antibiotic-induced dysbiosis [12], can intervene in the antitumoral protective response [13] and can have antiviral activity [14].

Of great interest is *Enterococcus faecium*, for which the European Food Safety Authority (EFSA) has recently established new guidelines for distinguishing between beneficial or potentially pathogenic strains based on their susceptibility to ampicillin and on the presence of specific genetic markers of virulence (esp, hylEfm, IS16).

It has been demonstrated that the culture supernatant of the *E. faecium* strain in the human intestinal epithelial cells has a strong bactericidal effect on enteroaggregative *Escherichia coli*, including the induction of membrane damage and cell lysis [15]. The ability of these bacteria to produce enterocins is remarkable, and these instead can be applied as food biopreservatives [16, 17]. In fact, *E. faecium* RZS C5, a natural cheese isolate, has a strong activity against *Listeria monocytogene* adhesion and invasion of Caco-2 cells [18].

*E. faecium* SF68® (NCIMB 10415) is present in pharmaceutical preparations as a feed additive for different animals [19, 20], since it is capable of lowering the bacterial concentration of *E. coli* and stimulates an anti-inflammatory response [21].

Therefore, the human intestinal microbiota contributes to maintaining the integrity and impermeability of the intestinal wall, which represents the first line of defense against pathogens. Among these, *Salmonella enterica* serovar t*yphimurium* (*S. typhimurium*) is one of the most common nontyphoidal *Salmonella* (NTS) considered a major cause of acute food infection [22]. This Gram-negative bacillus can cause severe diarrhoea, vomiting, fever, and death in severe cases, especially in children, the elderly, and immunocompromised patients.

*S. typhimurium* can survive and replicate within host macrophages and induces the activation of NF-kB and the secretion of proinflammatory cytokines, such as interleukin- (IL-) 8 [23] and tumor necrosis factor alpha (TNF-*α*) [24]. This inflammation also helps it to compete with the microorganisms of the host microbiota [25].

Probiotics attenuate NF-kB activation and inflammatory cytokine production in the intestinal epithelial cells *in vitro* [26, 27] and *in vivo* [28–30].

In addition to serving as a protective barrier, the intestinal epithelium plays an active role in the intestinal immune response through the secretion of inflammatory cytokines, chemokines, and antimicrobial peptides such as *β*-defensins [31].

The family of *β*-defensins is composed of small cationic peptides produced by epithelial cells, Paneth cells, neutrophils, and macrophages, constitutive or induced by microorganisms or cytokines that contribute to the broad spectrum innate immunity.

Human *β*-defensin-2 (hBD-2) is an inducible antimicrobial peptide with a molecular mass of 4–6 kD and acts as an endogenous antibiotic in the defense against Gram-negative bacteria, among which the potential pathogenic microbes of the gut [32, 33], and can be induced by endogenous stimuli, infections, or wounds.

Human *β*-defensin-3 (hBD-3) is identified in psoriatic scales [34] and is expressed in the skin, placenta, and oral tissue [34, 35] and shows antimicrobial activity against Gram-positive and Gram-negative bacteria and fungi. Being insensitive to high salt concentrations, its antimicrobial activity results to be greater than that of hBD-2 [36].

Both hBD-2 and hBD-3 are chemoattractants for neutrophils [37] and memory T-cells, induce histamine release from mast cells and prostaglandin synthesis, and play a role also in allergic responses.

In the light of the growing interest of the use of antimicrobial peptides as natural defense molecules against pathogens and due to the increased antibiotic resistance by a number of pathogenic bacteria, this study aims to create a line of intestinal epithelial cells expressing high concentrations of the antimicrobial peptides hBD-2 and hBD-3 and to assess their role in the host inflammatory response resulting from bacterial infections.

## 2. Materials and Methods

### 2.1. Cloning

Total RNA was extracted using a High Pure RNA Isolation Kit (Roche Diagnostics) from primary cultures of human keratinocytes stimulated with the LPS of *Pseudomonas aeruginosa* and TNF-*α* in order to obtain a high production of antimicrobial peptides. It was subsequently transcribed into complementary cDNA using random hexamer primers (Random hexamers, Roche) at 42°C for 45 minutes, according to the manufacturer's instructions. Two pairs of degenerate primers, designed on their specific amino acid sequence (hBD-2 for 5′-CCAGCCATCAGCCATGAGGGT-3′, hBD-2 rev-5′-GGAGCCCTTTCTGAATCCGCA-3′ 254 bp; and hBD-3 for 5′-CGGCAGCATTTTGCGCCA-3′, hBD-3 rev 5′-CTAGCAGCTATGAGGATC-3′), were used to amplify, by RT-PCR, gene coding hBD-2 and hBD-3 with FastStart High Fidelity (Roche Diagnostics). The amplification programs were the following: 35 cycles at 94°C for 1′, 63°C (for hBD-2) or 58°C (for hBD-3) for 1′, and 72°C for 1′; the PCR products were 254 and 206 base pairs.

The amplified DNA fragments were subjected to restriction and sequencing analysis and cloned into the pEF/V5-HIS TOPO (Invitrogen) vector using the T4 DNA ligase (Invitrogen), in accordance with the manufacturer's protocol, and then transformed into *E. coli* TOP 10 (Invitrogen).

The cloning vectors, pEF/V5-HIS TOPO-hBD-2 and pEF/V5-HIS TOPO-hBD-3, were extracted from the bacterial culture and amplified using a QIAprep Spin Midiprep Kit (QIAGEN).

### 2.2. Transfection

Caco-2 cells were transfected using the IBAfect reagent (IBA), according to the manufacturer's manuals. Briefly, 3 × 10^5^ cells were seeded in 6-well plates, and immediately after seeding, plasmids conjugated with the transfection reagent were added. The mixture was incubated for 24 and 48 hours. After incubation, the success of the experiment was verified by the extraction of mRNA from treated cells and by the amplification of hBD-2 and hBD-3 genes by PCR.

Cell-free supernatants of the transfected cells were recovered by centrifugation and assayed for the hBD-2 and hBD-3 concentration by an enzyme-linked immunosorbent assay (Phoenix Pharmaceuticals Inc.).

For blasticidin selection, untransfected and transfected cells were cultured at 37°C and 5% CO_2_ for 14 days in the presence of the following increasing concentrations of blasticidin S (Sigma-Aldrich): 5, 10, 20, 50, 100, and 200 *μ*g/ml. Then, MTT-labelling reagent was added at a final concentration of 0.5 mg/ml. After 4 hours, a solubilization solution was added to each well and the plates were incubated overnight. Spectrophotometric absorbance was measured using a microplate (ELISA) reader at a wavelength of 570 nm.

### 2.3. Bacterial Strains

*S. enterica* subsp*. enterica* serovar *typhimurium* (ATCC® 14028GFP™) was cultured on Luria-Bertani agar (Oxoid, Unipath, Basingstoke, UK). *E. faecium* (ATCC 27270™) was cultured on Bacto Tryptic Soy agar (TSA, Difco Laboratories). These strains were grown at 37°C for 18 h.

### 2.4. Cell Culture and Infection

Caco-2 cells (human Caucasian colon adenocarcinoma cells) were routinely cultured in Dulbecco's modified eagle medium (DMEM, Gibco) supplemented with 1% Penstrep, 1% glutamine, and 10% fetal calf serum (Invitrogen) at 37°C at 5% CO_2_. After transfection, the cells were grown in a sterile 75 cm^2^ flask at a concentration of 3 × 10^5^ to confluence for 21 days to reach full differentiation and polarization. The culture medium was changed every two days.

Subsequently, fully differentiated cells were seeded into six-well plates and then infected with exponentially growing bacteria at a multiplicity of infection (MOI) of 100 for 6 hours (for gene expression analysis) and 24 h (for ELISA assay) at 37°C in 5% CO_2_ in DMEM without antibiotics. In the case of coinfection, preincubation of one hour with *E. faecium* was followed by the addition of *S. typhimurium* without the removal of the probiotic bacterium.

At the end of the experiment, bacteria present in the supernatants of infected and coinfected cells were counted (CFUs) by spreading serial dilutions on selective medium HiCrome™ *E. faecium* Agar Base (Sigma-Aldrich) and Brilliance *Salmonella* agar (OXOID) and were incubated at 37°C overnight.

### 2.5. Real-Time PCR

In order to evaluate the expression of pro- and anti-inflammatory cytokines, the cells at the end of treatments were washed three times with sterile PBS, and the total RNA was extracted using High Pure RNA Isolation Kit (Roche Diagnostics).

Two hundred nanograms of total cellular RNA were reverse transcribed (Expand Reverse Transcriptase, Roche) into complementary DNA (cDNA) using random hexamer primers (Random hexamers, Roche) at 42°C for 45 minutes, according to the manufacturer's instructions [38]. Real-time PCR for IL-6, IL-8, TNF-*α*, IL-1*α*, IL-1*β*, and TGF-*β* was carried out with the LC FastStart DNA Master SYBR Green kit using 2 *μ*l of cDNA, corresponding to 10 ng of total RNA in a 20 ml final volume, 3 mM MgCl2, and 0.5 mM sense and antisense primers (Table 1). After amplification, melting curve analysis was performed by heating to 95°C for 15 s with a temperature transition rate of 20°C/s, cooling to 60°C for 15 s with a temperature transition rate of 20°C/s, and then heating the sample at 0.1°C/s to 95°C. The results were then analyzed using LightCycler software (Roche Diagnostics). The standard curve of each primer pair was established with serial dilutions of cDNA. All PCR reactions were run in triplicate. The specificity of the amplification products was verified by electrophoresis on a 2% agarose gel and visualization by ethidium bromide staining.

### 2.6. ELISA Assay for Pro- and Anti-Inflammatory Cytokines

Caco-2 cell monolayers were infected with *S. typhimurium* and/or *E. faecium* for 24 h at 37°C, as described above. At the end of the experiment, supernatants were harvested and the presence of cytokines IL-6, IL-8, IL-1*β*, TNF-*α*, and TGF-*β* was analyzed by enzyme-linked immunosorbent assay (ELISA, ThermoFischer Scientific Inc.).

### 2.7. Bacterial Internalization Assay

Untransfected Caco-2 cell cultures were infected with *S. typhimurium* alone or coinfected with *S. typhimurium* and *E. faecium* as previously described. In another set of experiments, *E. faecium* was heat killed by incubating at 60°C for 45 min and subcultured on TSA plates (Difco Laboratories) overnight at 37°C to prove that no viable organisms remained. Killed bacterial preparation was resuspended in DMEM without antibiotics and added to cell monolayer an hour before the addition of *S. typhimurium*. After 2 h of incubation at 37°C, infected monolayers were extensively washed with sterile PBS and further incubated for another two hours in the DMEM medium, and supplemented with gentamicin sulphate (250 *μ*g ml-1) (Sigma-Aldrich) in order to kill the extracellular bacteria. At the end of the experiments, infected monolayers were extensively washed in PBS then lysed with a solution of 0.1% Triton X-100 (Sigma-Aldrich) in PBS for 10 minutes at room temperature to count internalized bacteria. Aliquots of cell lysates were serially diluted and plated on Brilliance Salmonella agar (OXOID) and incubated at 37°C overnight to quantify viable intracellular bacteria (CFUs/ml). The efficiency was calculated as the ratio of the number of cell-internalized bacteria with the number of bacteria used to infect the cell monolayers.

### 2.8. Statistical Analysis

Significant differences among groups were assessed through two-way ANOVA by using GraphPad Prism 6.0. The data are expressed as means ± standard deviation (SD) of three independent experiments.

## 3. Results

### 3.1. Cloning and Transfection

The hBD-2 and hBD-3 genes were successfully amplified by RT-PCR from a total cellular RNA. As expected, the PCR products were 254 and 206 bp in length. These products were inserted with high efficiency in the pEF/V5-HIS TOPO vector.

**Figure 1:**
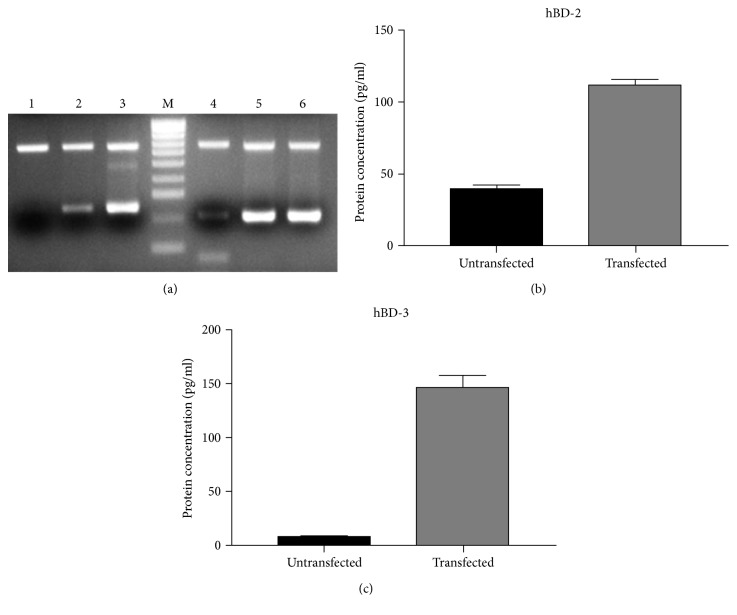
(a) hBD-2 mRNA expression in untransfected cells (lane 1), 24 hours (lane 2), and 48 hours (lane 3) after transfection; hBD-3 mRNA expression in untransfected cells (lane 4), 24 hours (lane 5), and 48 hours (lane 6) after transfection. (b) hBD-2 concentration in cell supernatants 48 hours after transfection. (c) hBD-3 concentration in cell supernatants 48 hours after transfection.

The success of transfection of the cloning products in colorectal adenocarcinoma Caco-2 cells was verified after 24 and 48 hours by RT-PCR and after 48 hours by ELISA assay on cell supernatants (Figure 1).

### 3.2. Blasticidin Selection and Cellular Viability

The toxicity curve performed on transfected and untransfected cells showed that the optimal antibiotic concentration for the selection of stable clones was 200 *μ*g/ml. These data were also supported by the results of the cellular viability assay (see Supplementary Material available online at https://doi.org/10.1155/2017/6976935). The selected clones were then cultured for an additional 21 days to obtain their differentiation, which was characterized by polarization and the formation of microvilli.

### 3.3. Evaluation of the Host Inflammatory Response

After the infection of untransfected and hBD-2- or hBD-3-transfected Caco-2 cells with *E. faecium* and/or *S. typhimurium*, we examined the host response by evaluating the expression of proinflammatory cytokines IL-6, IL-8 Il-1*α*, and IL-1*β* and anti-inflammatory cytokine TGF-*β* by real-time PCR.

**Figure 2:**
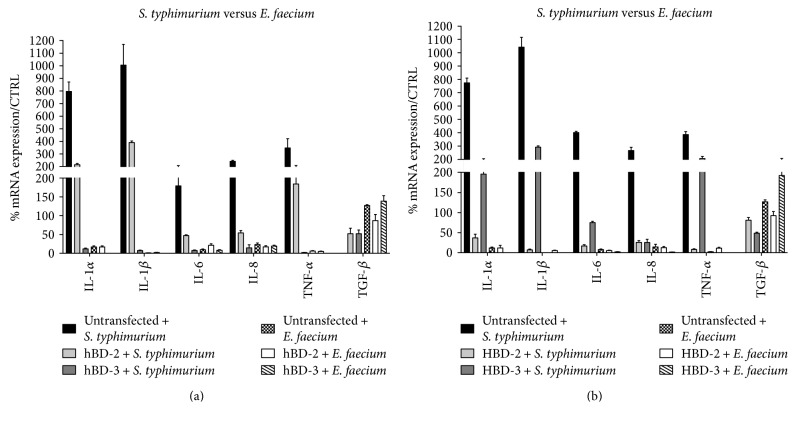
Comparison between relative gene expression (a) and protein concentration (b) in Caco-2 cells infected with *S. typhimurium* and Caco-2 cells infected with *E. faecium*. Data are mean ± SD and are expressed as the percentage of increment compared to uninfected controls.

The data obtained showed that the cells transfected with the hBD-2 and hBD-3 genes and infected with *S. typhimurium* showed a lesser expression of proinflammatory cytokines compared to the untransfected control. Instead, an infection of Caco-2 cells with *E. faecium* resulted only in a slight increase of expression of proinflammatory cytokines and an increase in anti-inflammatory cytokine TGF-*β*, which was more apparent in the presence of antimicrobial peptides; these data confirm that *E. faecium* did not act as a pathogen and did not induce an increase in the inflammatory response (Figure 2).

**Figure 3:**
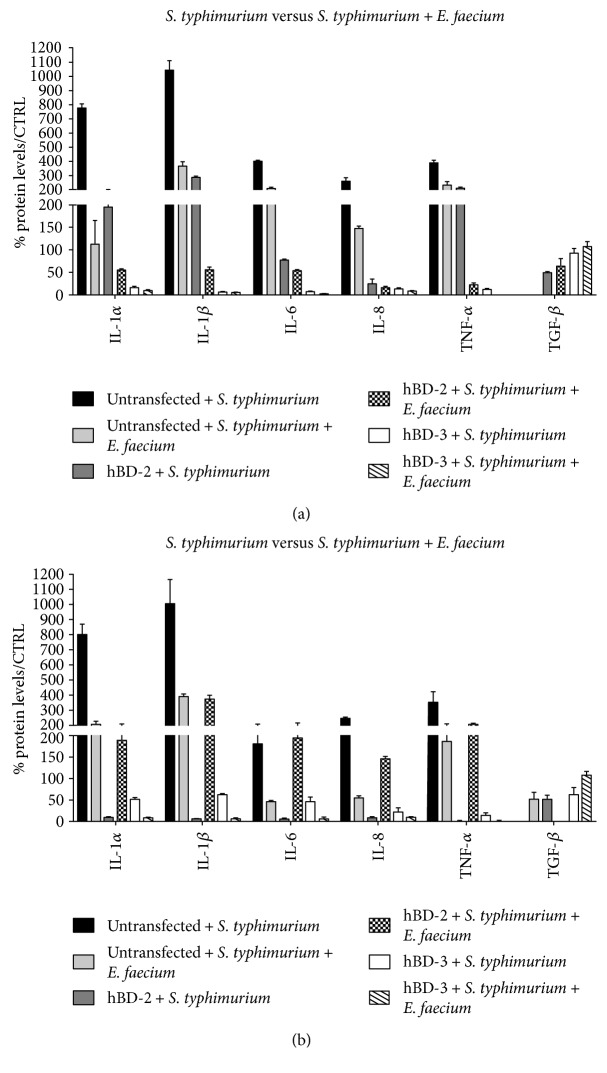
Comparison between relative gene expression (a) and protein concentration (b) in Caco-2 cells infected with *S. typhimurium* alone and Caco-2 cells coinfected with *S. typhimurium* and *E. faecium*. Data are mean ± SD and are expressed as the percentage of increment compared to uninfected controls.

In addition, during the coinfection with *S. typhimurium* and *E. faecium*, the already significant decrease in expression levels of proinflammatory cytokines revealed in the transfected cells during infection with *S. typhimurium* alone is even more pronounced, indicating that the antimicrobial peptides have enhanced probiotic antibacterial activity (Figure 3).

These data were also confirmed by ELISA protein assay.

### 3.4. Evaluation of Bacteria Viability

In order to test the toxicity of antimicrobial peptides against *S. typhimurium* and *E. faecium*, the supernatants of the coinfected cells were subjected to serial dilutions and plated on selective media.

Our results indicate that both hBD-2 and hBD-3 possess selective toxicity towards *S. typhimurium* and did not interfere with the growth of *E. faecium* (Table 2).

### 3.5. Effect of *E. faecium* and AMPs on *S. typhimurium* Invasiveness

**Figure 4:**
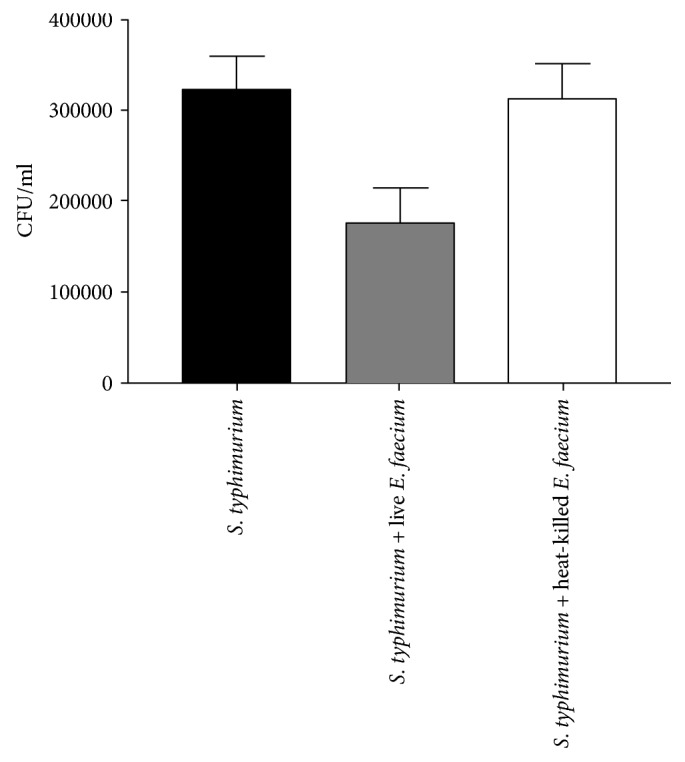
*S. typhimurium* internalization assay. Untransfected Caco-2 cells were infected with *S. typhimurium* alone or coinfected with live or heat-killed *E. faecium* for 4 hours. The number of internalized bacteria was determined by host cell lysis, plating, and counting CFU/well. The data shown are representative of three different experiments ± SD. Error bars represent standard deviations.

Preincubation of untransfected Caco-2 cells with live *E. faecium* significantly affected *S. typhimurium* internalization, reducing it by 45.8%. Conversely, pretreatment with heat-killed *E. faecium* does not interfere with the invasive capacity of the pathogen (Figure 4).

## 4. Discussion

Innate immunity, in particular through antimicrobial peptides (AMPs), plays a key role in maintaining the balance between protection against pathogens and normal microbial tolerance; AMPs are structurally heterogeneous peptides of amphipathic nature isolated from a wide variety of organisms, plants, insects, amphibians, and mammals that are able to kill bacteria, fungi, and viruses quickly. Among these, the human *β*-defensins have received considerable interest. These peptides are produced by epithelial cells, constitutively, or as a result of certain stimuli such as microorganisms or cytokines. Defensins are able to attract inflammatory cells such as neutrophils, T cells, macrophages, and epithelial cells capable of releasing inflammatory mediators such as IL-6, IL-8, and IL-1*β*, as well as destabilizing microbial membranes; moreover, they have the ability to remodel the tissues and bind LPS.

In particular, *β*-defensin-2 (hBD-2) and *β*-defensin-3 (hBD-3) are present in various epithelia, such as skin [39], oral cavity [40], paranasal sinuses, gingival [41], corneal [42], intestinal, respiratory, and urogenital epithelium [31], and show antimicrobial activity against Gram-positive and Gram-negative bacteria and fungi.

It has been estimated that the number of microbes present throughout the human body amounts to approximately 100 trillion cells, tenfold the number of human cells, and suggested that they encode 100-fold more unique genes than our own genome [43]. Most of them are components of the gut microbiota, which contains between 1000 and 1150 prevalent bacterial species that play a central role in human health [43, 44].

This community is defined as a “metabolic organ,” as it plays a primary role in maintaining homeostasis by intervening in the regulation of metabolism and nutritional, physiological, and immunological functions.

In the first phase of this work, we worked on creating, by cloning and gene transfection techniques, a line of intestinal epithelial cells (Caco-2 cells) that expresses hBD-2 and hBD-3 genes. This allowed us to evaluate the role of these peptides in protecting the intestinal epithelium against *S. typhimurium*, alone or in cooperation with *E. faecium*, which is one of the major components of the human gut microbiota [45, 46].

The first data obtained from the CFUs/ml counts following infection and coinfection showed that there was a marked reduction in the number of colonies of *S. typhimurium* compared to untransfected cells in transfected cells, while the number of colonies of *E. faecium* remained unchanged, which shows that the antimicrobial peptides selectively carried out their microbicidal activity against the pathogen.

Mucosal surfaces are lined with epithelial cells that form a barrier between potentially pathogenic microorganisms and the host tissues. Penetration of this layer by invasive bacteria initially leads to an acute inflammatory response, a hallmark of which is the local accumulation of polymorphonuclear leukocytes. After the infection with pathogenic bacteria, epithelial cells at mucosal surfaces can secrete chemical mediators, such as proinflammatory and chemoattractant cytokines constituting surveillance and warning system for the immune and inflammatory cells present in the underlying mucosa [47]. However, in sites where there is a physiologically high bacterial concentration due to the resident microbial flora, that is, the colon, cytokine production is closely dependent on bacterial invasiveness, as only invasive bacteria induce cytokine secretion [48–50].

Among these, IL-6, IL-1, and TNF-*α* are highly expressed in most inflammatory states so as to often be considered a target of therapeutic intervention.

IL-8 chemokine is also thought to be an early signal of acute inflammation, as it is secreted by the intestinal epithelial cells following bacterial invasion, and accumulates in the mucosa underlying the epithelial cell layer where the IL-8 responsive effector cells reside. In addition, it has been shown that the presence of the IL-8 in serum is a diagnostic marker for neonatal bacterial infection [51, 52].

The results obtained show that the inflammatory response in hBD-2- and hBD-3-transfected cells is modified with respect to untransfected cells, since the expression of proinflammatory cytokines IL-6, IL-8, TNF-*α*, IL-1*α*, and IL-1*β* is greatly reduced, while the expression of anti-inflammatory cytokine TGF-*β* is increased. These data indicate that the invasive and inflammatory potential of *S. typhimurium* is significantly reduced in the presence of antimicrobial peptides.

Experiments of coinfection of untransfected cells with *S. typhimurium* and probiotic *E. faecium* showed that in the presence of *E. faecium*, *Salmonella* infection caused a much less intense inflammatory response, and this data is confirmed by invasive assays in which the presence of *E. faecium* results in a reduction in the internalization of *S. typhimurium* by 45.8%. However, the more interesting result is that the decrease in the level of inflammatory response due to the presence *of E. faecium* is further reduced in the transfected cells, that is, in the presence of high concentrations of antimicrobial peptides, suggesting that antimicrobial peptides may enhance the beneficial probiotic activity.

In our experimental system, the ability of AMPs to significantly reduce the inflammatory response in infected and coinfected cells is also due to their killing activity against *Salmonella*, as also demonstrated by the count of CFUs/ml following coinfection, in which the concentration of pathogen is considerably reduced in the presence of AMPs with respect to untransfected cells. AMPs could be considered, in the future, as a new class of therapeutics since they are able to induce lesser resistance and have a selective antimicrobial activity to protect the host without the need for the immune system memory [53]. Having an *in vitro* system that will produce these proteins will allow us to better clarify the mechanisms underlying these different behaviors.

## Supplementary Material

The table shows the values of O.D. of transfected and untransfected cells in the presence of increasing blasticidine concentrations.

## Figures and Tables

**Table 1:** Primer sequences and amplification programs.

Gene	Primer sequence	Conditions	Product size (bp)
IL-6	5′-ATGAACTCCTTCTCCACAAGCGC-3′ 5′-GAAGAGCCCTCAGGCTGGACTG-3′	5^″^at 95°C, 13^″^ at 56°C, and 25^″^at 72°C for 40 cycles	628
IL-8	5′-ATGACTTCCAAGCTGGCCGTG-3′ 5′-TGAATTCTCAGCCCTCTTCAAAAACTTCTC-3′	5^″^at 94°C, 6^″^ at 55°C, and 12^″^at 72°C for 40 cycles	297
IL-1*β*	5′-GCATCCAGCTACGAATCTCC-3′ 5′-CCACATTCAGCACAGGACTC-3′	5^″^at 95°C, 14^″^ at 58°C, and 28^″^at 72°C for 40 cycles	708
TGF-*β*	5′-CCGACTACTACGCCAAGGAGGTCAC-3′ 5′-AGGCCGGTTCATGCCATGAATGGTG-3′	5^″^at 94°C, 9^″^ at 60°C, and 18^″^at 72°C for 40 cycles	439
IL-1*α*	5′-CATGTCAAATTTCACTGCTTCATCC-3′ 5′-GTCTCTGAATCAGAAATCCTTCTATC-3′	5^″^at 95°C, 8^″^at 55°C, and 17^″^at 72°C for 45 cycles	421
TNF-*α*	5′-CAGAGGGAAGAGTTCCCCAG-3′ 5′-CCTTGGTCTGGTAGGAGACG-3′	5^″^at 95°C, 6^″^ at 57°C, and 13^″^at 72°C for 40 cycles	324

**Table 2:** CFUs/ml of *S. typhimurium* and *E. faecium* in supernatants of coinfected cells.

	Inoculum	Untransfected	hBD-2-transfected	hBD-3-transfected
*S. typhimurium*	1 × 10^7^	2 × 10^6^	5 × 10^4^	4,3 × 10^3^
*E. faecium*	3 × 10^8^	3 × 10^8^	3 × 10^8^	2 × 10^8^
