# Supplementary figures and images for: Beta-Defensin-2 and Beta-Defensin-3 Reduce Intestinal Damage Caused by Salmonella typhimurium Modulating the Expression of Cytokines and Enhancing the Probiotic Activity of Enterococcus faecium
Source: J Immunol Res. 2017 Nov 9;2017:6976935. doi: 10.1155/2017/6976935 (PMC5700477; doi:10.1155/2017/6976935)

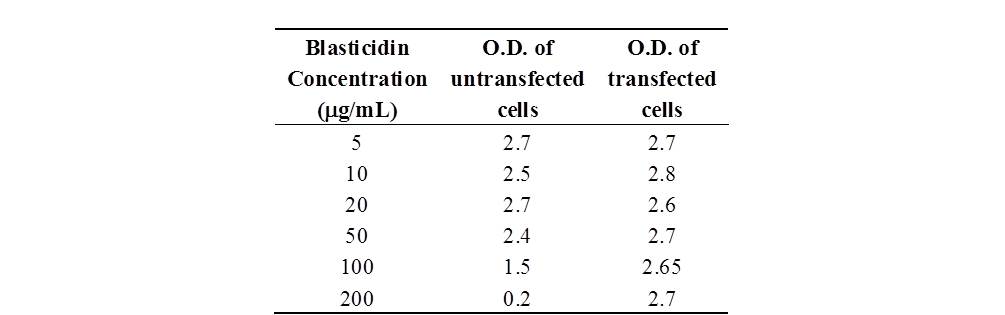

Supplement: Supplementary file 1 — The table shows the values of O.D. of transfected and untransfected cells in the presence of increasing blasticidine concentrations. [file 6976935.f1.jpg]
